# Infections, antibiotic treatment and mortality in patients admitted to ICUs in countries considered to have high levels of antibiotic resistance compared to those with low levels

**DOI:** 10.1186/1471-2334-14-513

**Published:** 2014-09-22

**Authors:** Håkan Hanberger, Massimo Antonelli, Martin Holmbom, Jeffrey Lipman, Peter Pickkers, Marc Leone, Jordi Rello, Yasser Sakr, Sten M Walther, Philippe Vanhems, Jean-Louis Vincent, for the EPIC II Group of Investigators

**Affiliations:** Division of Infectious Diseases, Institution of Clinical and Experimental Medicine, Faculty of Health Sciences, Linköping University, Linköping, Sweden; Università Cattolica del Sacro Cuore - Policlinico Universitario A. Gemelli, Rome, Italy; Department of Intensive Care Medicine, Royal Brisbane and Women’s Hospital, The University of Queensland, Herston, QLD Australia; Department of Intensive Care Medicine, Radboud University Medical Centre, Nijmegen, The Netherlands; Department of Intensive Care and Anesthesiology, Hôpital Nord, AP-HM Unité de Recherche en Maladies Infectieuses et Transmissibles (URMITE), Aix-Marseille University, Marseilles, France; Critical Care Department, Vall d'Hebron University Hospital, CIBERES, VHIR, Universitat Autonoma de Barcelona, Barcelona, Spain; Department of Anesthesiology and Intensive Care, Friedrich-Schiller University, Jena, Germany; Division of Cardiovascular Medicine, Department of Medicine and Health, Faculty of Health Sciences, Linköping University, Linköping, Sweden; Service d’Hygiène, Epidémiologie et Prévention, Groupement Hospitalier Edouard Herriot, Hospices Civils de Lyon, Université Lyon 1, Lyon, France; Department of Intensive Care, Erasme Hospital, Université Libre de Bruxelles, Brussels, Belgium

**Keywords:** Infection, Critically ill, Antibiotic, Resistance

## Abstract

**Background:**

Antimicrobial resistance is an increasing concern in ICUs worldwide. Infection with an antibiotic resistant (ABR) strain of an organism is associated with greater mortality than infection with the non-resistant strain, but there are few data assessing whether being admitted to an intensive care unit (ICU) with high levels of antimicrobial resistance is associated with a worse outcome than being admitted to an ICU with low rates of resistance. The aim of this study was, therefore, to compare the characteristics of infections and antibiotic treatments and patient outcomes in patients admitted to ICUs in countries considered as having high levels of antibiotic resistance and those admitted to ICUs in countries considered as having low levels of antibiotic resistance.

**Methods:**

Data from the large, international EPIC II one-day point prevalence study on infections in patients hospitalized in ICUs were used. For the current study, we compared the data obtained from patients from two groups of countries: countries with reported MRSA rates of ≥ 25% (highABR: Greece, Israel, Italy, Malta, Portugal, Spain, and Turkey) and countries with MRSA rates of < 5% (lowABR: Denmark, Finland, Netherlands, Norway, and Sweden).

**Results:**

On the study day, 1187/2204 (53.9%) patients in the HighABR ICUs were infected and 255/558 (45.7%) in the LowABR ICUs (P < 0.01). Patients in the HighABR ICUs were more severely ill than those in the LowABR ICUs, as reflected by a higher SAPS II score (35.6 vs 32.7, P < 0.05) and had longer median ICU (12 days vs 5 days) and hospital (24 days vs 16 days) lengths of stay. They also had higher crude ICU (20.0% vs 15.4%) and hospital (27.0% vs 21.5%) mortality rates (both P < 0.05). However, after multivariable adjustment and matched pair analysis there were no differences in ICU or hospital mortality rates between High or LowABR ICU patients overall or among those with infections.

**Conclusions:**

Being hospitalized in an ICU in a region with high levels of antimicrobial resistance is not associated per se with a worse outcome.

**Electronic supplementary material:**

The online version of this article (doi:10.1186/1471-2334-14-513) contains supplementary material, which is available to authorized users.

## Background

The prevalence of infection is high among patients admitted to ICUs and is one of the main causes of ICU mortality [1, 2]. In 1992, a one-day European point-prevalence study on infections in intensive care (EPIC), performed in 1417 ICUs in 17 Western European countries [3], showed that approximately 45% of all patients present on the ICU on the study day were infected. The mean prevalence of ICU-acquired infections was 21%, but varied widely from 10% in Switzerland to 32% in Italy [3]. EPIC II was conducted 15 years later, on May 8, 2007 [2]. Data were collected from 13796 adult (>18 years) patients from 1265 ICUs in 75 countries and showed that 51% of patients had at least one infection.

Antimicrobial resistance is an increasing concern in ICUs worldwide. Rates of resistance vary considerably across different countries and regions; for example, ICUs in southern Europe generally have higher rates of resistance than countries in northern Europe and Scandinavia [4]. Several studies have reported that infection with an antibiotic resistant strain of an organism is associated with greater mortality and costs and longer ICU lengths of stay than infection with the non-resistant strain [5–9]. An important question is whether being admitted to an ICU with high levels of antimicrobial resistance is associated with a worse outcome than being admitted to an ICU with low rates of resistance, but there are few data available on this topic. In the present study, therefore, we used data from the large EPIC II study [2] to compare the characteristics of infections, antibiotic treatments and patient outcomes in patients admitted to ICUs in countries considered as having high levels of antibiotic resistance, as recorded in the Antimicrobial Resistance Surveillance System (EARSS) 2007 Annual Report, and those admitted to ICUs in countries considered as having low levels of antibiotic resistance. We hypothesized that there would be a difference in outcomes in patients admitted to ICUs in countries with high levels of antimicrobial resistance compared to those admitted to ICUs in countries with low levels of resistance.

## Methods

The worldwide EPIC II 1-day point-prevalence study of infection and demographics of critically ill patients was performed on 8 May 2007 [2]. Data were collected for 13,796 adult patients in 1265 participating ICUs from 75 countries on the study day. Infection was defined according to the criteria of the International Sepsis Forum [10]. Microbiological analyses were performed locally. Participating ICUs were asked to provide patient follow-up until hospital discharge or for 60 days.

For the purposes of this analysis, we selected two groups of countries based on the country rate of methicillin-resistant *Staphylococcus aureus* (MRSA) reported in the European Antimicrobial Resistance Surveillance System (EARSS) 2007 Annual Report [11]: countries with MRSA rates of ≥ 25% in the 2007 report (Greece, Israel, Italy, Malta, Portugal, Spain, and Turkey) and countries with MRSA rates of < 5% (Denmark, Finland, Netherlands, Norway, and Sweden) (see Appendix for list of participating centres in these countries). Severity scores, source of infection, pathogens, antibiotic resistance, antibiotic therapy (prophylactic and therapeutic, as defined by the attending physician at each centre), co-morbidities, lengths of stay and outcomes were compared in patients in the two groups of countries.

The EPIC II study was approved by the ethics committee of Erasme Hospital, Belgium, the coordinating center. Local ethical committee approval at each participating center (see Appendix for list of participating centers) was expedited or waived because of the observational nature of the study.

### Statistical analyses

Statistical analyses were performed using IBM SPSS Statistics 20 for Windows (IBM, Armonk, NY, USA). The Kolmogorov-Smirnov test was used, and histograms and Q-Q plots were examined to verify if there were significant deviations from the normality assumption of continuous variables. Difference testing between groups was performed using Mann-Whitney test, *χ*^2^ test, or Fisher’s exact test as appropriate. Logistic regression analysis was used to determine the mortality risk associated with admission to an ICU in a country with high levels of antimicrobial resistance or one with low levels of antimicrobial resistance. To remove any bias of confounding variables for the association between the areas of ICU admission and mortality, a propensity score was estimated for each of the ICU areas, with the following variables considered as factors: type of admission, source of admission, comorbidities, age, sex, mechanical ventilation, haemofiltration or haemodialysis, infection, SAPS II score and type of microorganism. After checking that balance on all covariates that were used in the propensity model had been achieved, odds ratios with mortality as the dependent variable were estimated by logistic regression using two strategies: by matching and by introducing the propensity score in the model [12, 13]. Reported odds ratios were adjusted for propensity score, hospital and organizational related factors, including type of ICU (closed vs. open, community vs. university, surgical vs. medical), number of ICU beds, number of nurses, number of physiotherapists, presence of 24-h ICU physician coverage, length of ICU stay prior to study day, percentage of gross domestic product spent on health care generated using the World Health Organization Statistical Information System (based on data from 2006 [14]) and geographical region. Data are presented as median [interquartile range (IQR)] or number (%), as appropriate. All tests were two-tailed, and a P < 0.05 was considered statistically significant.

## Results

In the EPIC II database, there were 2204 patients included from ICUs in countries with high levels of antimicrobial resistance (HighABR) according to the EARSS 2007 Annual Report [11] and 558 from ICUs in countries with low levels of antimicrobial resistance (LowABR) Table 1). The median SAPS II score was significantly higher in HighABR than in LowABR ICU patients (34 [26-44] vs 31 [25-39] P < 0.001) and chronic renal failure and chronic obstructive pulmonary disease (COPD) were significantly more common (Table 1). Patients from HighABR ICUs were more often admitted from the emergency room and less often from the operating/recovery room than were patients from LowABR ICUs. Patients from HighABR ICUs had longer ICU and hospital lengths of stay than patients from LowABR ICUs; the number of days in the ICU prior to the study day was also longer in patients admitted to HighABR ICUs (Table 1).

**Table 1:** **Characteristics of study population and participating units**

Characteristics		LowABR countries	HighABR countries	p-value
Of ICUs		N = 54	N = 231	
Staffed ICU beds, n (%)				
<7		14 (25.9)	67 (29.0)	0.9
7-14		30 (55.6)	122 (52.8)	
15+		10 (18.5)	42 (18.2)	
Nurse:patient, n (%)				
>1:1		19 (35.2)	16 (6.9)	<0.001
1:1-1:1.49		14 (25.9)	42 (18.2)	
1:1.5-1:1.99		18 (33.3)	78 (33.8)	
<1:2		3 (5.6)	95 (41.1)	
24-hr in-house intensivist		48 (88.9)	231 (100%)	<0.001
**Of patients**		**N = 558**	**N = 2204**	
Age, median (25-75 percentile)		65 (53-74)	65 (51-74)	0.574
SAPS II score, median (25-75 percentile)		31 (25-39)	34 (26-44)	<0.001
SOFA score, median (25-75 percentile)		5 (3-8)	5 (3-8)	0.806
Length of stay in days in ICU prior to study day, median (25-75 percentile)		1 (0-6)	4 (0-14)	<0.001
Infected		255 (45.7)	1187 (53.9)	0.001
Type of admission, n (%)	*Surgical, elective*	203 (36.5)	455 (20.7)	<0.001
	*Surgical, emergency*	200 (36.0)	952 (43.2)	
	*Medical*	114 (20.5)	534 (24.3)	
	*Trauma*	39 (7.0)	261 (11.9)	
Admission source, n (%)	*OR/recovery*	205 (37.7)	472 (21.5)	< 0.001
	*ED/ambulance*	111 (20.4)	680 (31.0)	
	*Hospital ward*	160 (29.4)	713 (32.5)	
	*Other hospital*	61 (11.2)	273 (12.4)	
	*Other*	7 (1.3)	55 (2.5)	
Main reason for admission, n (%)	*Surveillance/monitoring*	158 (28.3)	336 (15.2)	< 0.001
	*Neurological*	56 (10.0)	350 (15.9)	
	*Respiratory*	119 (21.3)	527 (23.9)	
	*Cardiovascular*	119 (21.3)	579 (26.3)	
	*Renal*	5 (0.9)	33 (1.5)	
	*Digestive/liver*	53 (9.5)	154 (7.0)	
	*Trauma*	36 (6.5)	198 (9.0)	
	*Other*	12 (2.2)	27 (1.2)	
Co-morbidities, n (%)	*COPD*	77 (13.8)	457 (20.7)	<0.001
	*Haematological cancer*	11 (2.0)	49 (2.2)	0.715
	*Cancer*	82 (14.7)	341 (15.5)	0.649
	*IDDM*	42 (7.5)	221 (10.0)	0.072
	*Heart Failure (NYHA III-IV)*	59 (10.6)	231 (10.5)	0.949
	*CRF*	14 (2.5)	165 (7.5)	<0.001
	*HIV*	2 (0.4)	15 (0.7)	0.385
	*Cirrhosis*	14 (2.5)	65 (2.9)	0.577
	*Immunosuppression*	24 (4.3)	95 (4.3)	0.992
Number of co-morbidities, n (%)	*None*	282 (50.5)	1011 (45.9)	0.029
	*1*	195 (34.9)	746 (33.8)	
	*2*	61 (10.9)	313 (14.2)	
	*3*	16 (2.9)	103 (4.7)	
	*>3*	4 (0.7)	31 (1.4)	
Treatments, n (%)	*Mechanical ventilation*	359 (64.7)	1388 (63.1)	0.502
	*Haemofiltration or haemodialysis*	47 (8.5)	171 (7.8)	0.591
Length of stay, median (25-75 percentile)	ICU	5 (1-19)	12 (3-31)	< 0.001
	Hospital	16 (7-37)	24 (10-54)	<0.001
Mortality	ICU	83 (15.4)	408 (20.0)	0.016
	Hospital	116 (21.5)	551 (27.0)	0.01

OR, operating room; ED, emergency department; COPD, chronic obstructive pulmonary disease; IDDM, insulin-dependent diabetes mellitus; NYHA III-IV, New York Heart Association class III-IV; CRF, chronic renal failure; HIV, human immunodeficiency virus.

### Infections and Microbiology

More patients in the HighABR ICUs than in the LowABR ICUs were infected on the study day (53.9% vs 45.7%, P < 0.01). The respiratory system was the most common site of infection in all patients (Additional file 1). The sites of infection were similar in the two groups except for abdominal infections, which were more common in patients admitted to LowABR ICUs (24.3% vs 18.4%, P = 0.03), and bloodstream infections, which were more common in patients admitted to HighABR ICUs (19.6% vs 16.1%, P = 0.03). Culture specimens were positive in 852 (71.8%) of the patients from HighABR ICUs and in 196 (76.9%) of the patients from LowABR ICUs (P = NS). As expected, MRSA was more common in patients in the HighABR ICUs than those in the LowABR ICUs (69/137 [50%] vs 2/32 [6%], P < 0.001) (Table 2). Infections caused by *Pseudomonas aeruginosa* or *Actinetobacter* were significantly more prevalent among HighABR ICU patients (Table 2), whereas methicillin-sensitive *S. aureus* (MSSA), enterococci and anaerobes were more common in LowABR ICU patients.

**Table 2:** **Microorganisms isolated from the 1048 culture-positive infected patients**

	LowABR countries (n = 196)	HighABR countries (n = 852)	P-value
Pathogen, n (%)			
*S. aureus*	32 (16.3)	137 (16.1)	0.993
MRSA	2 (1.0)	69 (8.1)	<0.001
MSSA	30 (15.3)	70 (8.2)	0.002
*S. epidermidis*	18 (9.2)	117 (13.7)	0.087
*S. pneumoniae*	14 (7.1)	39 (4.6)	0.139
*Enterococci*	31 (15.8)	92 (10.8)	0.049
G^+^, others	20 (10.2)	48 (5.6)	0.019
*E. coli*	36 (18.4)	128 (15.0)	0.245
*Enterobacteriaceae*	10 (5.1)	52 (6.1)	0.592
*Klebsiella*	21 (10.7)	92 (10.8)	0.973
*P. aeruginosa*	27 (13.8)	172 (20.2)	0.039
*Acinetobacter spp*	5 (2.6)	130 (15.3)	<0.001
ESBL-producing	4 (2.0)	10 (1.2)	0.340
G^-^, others	37 (18.9)	136 (16.0)	0.322
Anaerobes	15 (7.7)	22 (2.6)	<0.001
Bacteria, others	3 (1.5)	12 (1.4)	0.897
*Candida*	45 (23.0)	156 (18.3)	0.136
*Aspergillus*	1 (0.5)	8 (0.9)	0.557
Fungi, others	5 (2.6)	6 (0.7)	0.022
Parasites	4 (2.0)	6 (0.7)	0.083
Organisms, others	12 (6.1)	19 (2.2)	0.004

MRSA, methicillin-resistant S. aureus; MSSA, methicillin-sensitive S. aurues; G^+^, Gram positive; ESBL, extended-spectrum-beta-lactamase; G-, Gram negative.

### Antibiotic use

#### Treatment

Use of therapeutic antimicrobials was significantly more common overall in HighABR ICU patients than in LowABR ICU patients (54.6% vs 44.4%, P < 0.001). When considering groups of antimicrobials, carbapenems, aminoglycosides, quinolones and glycopeptides were more commonly used in patients in HighABR ICUs than in LowABR ICUs, whereas cephalosporins were used more frequently as treatment in LowABR ICUs (Table 3). For individual antimicrobials, piperacillin-tazobactam, imipenem/meropenem, amikacin, oxazolidinone and tigecycline were used more frequently in patients in HighABR ICUs; cefazolin, cefuroxime, benzylpenicillin, oxa-/cloxa-/flucloxacillin, erythromycin and metronidazole were used more frequently in patients in LowABR ICUs (Table 3).

**Table 3:** **Use of antimicrobials as treatment in infected patients**

	LowABR ICU patients	HighABR ICU patients	p-value
	(n = 255)	(n = 1187)	
Antimicrobial, n (%)			
Cephalosporins (all)	84 (32.9)	206 (17.4)	<0.001
*Cefazolin*	4 (1.6)	4 (0.3)	0.016
*Cefuroxime*	46 (18.0)	2 (0.2)	<0.001
*Ceftazidime*	8 (3.1)	40 (3.4)	0.851
*Cefepime/Cefpirome*	1 (0.4)	24 (2.0)	0.070
*Other cephalosporins*	26 (10.2)	139 (11.7)	0.491
Penicillins (all)	84 (32.9)	400 (33.7)	0.816
*Benzylpenicillin*	16 (6.3)	3 (0.3)	<0.001
*Ampicillin*	5 (2.0)	29 (2.4)	0.645
*Amoxycillin + Clavulanic acid*	16 (6.3)	71 (6.0)	0.858
*Piperacillin + Tazobactam*	27 (10.6)	273 (23.0)	<0.001
*Oxa-/Cloxa-/Flucloxacillin*	14 (5.5)	16 (1.3)	<0.001
*Other penicillins*	8 (3.1)	20 (1.7)	0.127
Other β-lactams (all)	44 (17.3)	381 (32.1)	<0.001
*Imipenem/meropenem*	44 (17.3)	365 (30.7)	<0.001
*Aztreonam*	0 (0.0)	13 (1.1)	0.093
*Unspecified beta-lactams*	0 (0.0)	7 (0.6)	0.219
Aminoglycosides (all)	17 (6.7)	202 (17.0)	<0.001
*Amikacin*	0 (0.0)	114 (9.6)	<0.001
*Tobramycin*	11 (4.3)	26 (2.2)	0.052
*Other aminoglycosides*	6 (2.4)	66 (5.6)	<0.001
Quinolones (all)	38 (14.9)	242 (20.4)	0.045
*Ciprofloxacin*	31 (12.2)	128 (10.8)	0.525
*Other quinolones*	7 (2.7)	114 (9.7)	<0.001
Glycopeptides (all)	30 (11.8)	272 (22.9)	<0.001
*Vancomycin*	30 (11.8)	193 (16.3)	0.072
*Other glycopeptides*	0 (0.0)	81 (6.8)	<0.001
Macrolides (all)	13 (5.1)	48 (4.0)	0.448
*Erythromycin*	6 (2.4)	4 (0.3)	<0.001
*Other macrolides*	7 (2.7)	44 (3.7)	0.451
Other antibiotics (all)	55 (21.6)	321 (27.0)	0.071
*Metronidazole*	36 (14.1)	71 (6.0)	<0.001
*Cotrimoxazole*	5 (2.0)	30 (2.5)	0.594
*Oxazolidinone*	0 (0.0)	128 (10.8)	<0.001
*Tigecycline*	0 (0.0)	21 (1.8)	0.032
*Unspecified*	19 (7.5)	130 (11.0)	0.096
Antifungals (all)	52 (20.4)	209 (17.6)	0.295
*Fluconazole*	31 (12.2)	110 (9.3)	0.159
*Amphotericin B*	4 (1.6)	18 (1.5)	0.951
*Amphotericin lipid complex*	3 (1.2)	21 (1.8)	0.502
*Caspofungin*	12 (4.7)	40 (3.4)	0.299
*Voriconazole*	1 (0.4)	31 (2.6)	0.029
*Other antifungals*	1 (0.4)	2 (0.2)	0.477
Antivirals (all)	3 (1.2)	22 (1.9)	0.452

#### Prophylaxis

Use of prophylactic antimicrobials was significantly less common in HighABR ICU patients than in LowABR ICU patients (22.2% vs 32.8%, P < 0.001). Among the agents most commonly used were aminoglycosides (tobramycin), antifungals (amphotericin B) and cephalosporins (Additional file 2).

### Mortality

Crude ICU (20.0% and 15.4%) and hospital (27.0% vs 21.5%) mortality rates were higher in patients admitted to HighABR than in those admitted to LowABR ICUs (both P < 0.05); however, these differences were not present after adjustment in multivariable analysis or the matched pair analysis (Table 4). For subgroups of infected or non-infected patients there were no significant differences in crude or adjusted mortality rates between HighABR and LowABR ICUs.

**Table 4:** Crude and adjusted odds ratios* (95% CI) for ICU and hospital mortality in the whole cohort and in infected patients

	OR (95% CI)	P-value	m-OR (95% CI)	P-value	a-OR (95% CI)	P-value
ICU mortality (all)	1.37 (1.06-1.78)	0.016	1.14 (0.76-1.73)	0.526	1.07 (0.78-1.48)	0.661
Hospital mortality (all)	1.35 (1.07-1.69)	0.01	1.07 (0.74-1.55)	0.707	1.12 (0.84-1.48)	0.445
ICU mortality (infected)	1.23 (0.88-1.72)	0.222	1.23 (0.73-2.09)	0.438	0.99 (0.66-1.49)	0.95
Hospital mortality (infected)	1.21 (0.89-1.63)	0.218	1.02 (0.63-1.65)	0.933	1.1 (0.77-1.58)	0.606
ICU mortality (not infected)	1.38 (0.91-2.09)	0.131	1.01 (0.5-2.02)	0.984	1.32 (0.78-2.22)	0.304
Hospital mortality (not infected)	1.33 (0.93-1.92)	0.121	1.16 (0.64-2.11)	0.629	1.19 (0.75-1.9)	0.461

OR: Odds ratio; m-OR: Odds ratio from matched-pairs. a-OR: adjusted odds ratio. *With admission to HighABR ICU as the reference category. The list of confounders included in the multivariable analysis is reported in the text.

Patients with infections due to *Pseudomonas aeruginosa* had crude ICU and hospital mortality rates of 32.4% and 42.7%, respectively. Corresponding percentages for patients with *Acinetobacter* infections were 31.7 and 37.3, respectively, and for MRSA 32.3 and 43.1, respectively. Because of the relatively small sample sizes, we were unable to compare outcomes for the different pathogens in the two groups.

## Discussion

The main message of this study is that hospitalization in an ICU in a country with high levels of antimicrobial resistance is not an independent risk factor for worse outcome. We chose to select countries with likely high and low antimicrobial resistance using reported MRSA rates, although acknowledge that general resistance rates may have been different; nevertheless, most countries with high MRSA rates do seem to have high resistance levels among other organisms [11]. Several studies have shown increased mortality rates in patients with MRSA infections compared to those with MSSA infections [15, 16]. In an earlier analysis of the EPIC II database, MRSA infection was associated with an increased risk of death compared to MSSA infection (OR 1.46 [95% CI 1.03-2.06], P = 0.03) [17]. Using data from the European Antimicrobial Resistance Surveillance System and the Burden of Resistance and Disease in European Nations project, De Kraker et al. [15] reported that 27,711 episodes of MRSA blood stream infections (BSIs) were associated with 5,503 excess deaths. The same authors compared two prospective cohorts of patients from 13 ICUs in 13 European countries, one with MRSA and one with MSSA BSIs, each matched with control patients without the respective BSI [16]. MRSA and MSSA patients had higher 30-day and hospital mortality than the control patients, and MRSA patients had greater 30-day mortality (OR 1.8, P = 0.04) than MSSA patients. Other studies have reported similar mortality rates in MRSA and MSSA patients, but longer lengths of stays and higher associated costs [6, 7, 18].

Infections due to difficult to treat pathogens, i.e., *P. aeruginosa*, *Acinetobacter spp* and MRSA, were in general associated with higher crude ICU and hospital mortality rates in our study than other infections and were significantly more prevalent in HighABR ICUs; however, because of small sample sizes, comparisons between groups were not possible. We know from other ICU studies that these pathogens are associated with increased attributable mortality if empirical treatment is not appropriate [19–21]. Unfortunately, we are unable to determine whether or not treatment was appropriate in our study, although we did show that broadspectrum antibiotics, such as carbapenems, piperacillin-tazobactam, amikacin, tigecycline, oxazolidinone and vancomycin, were more commonly used for treatment in the HighABR ICUs. Similarly, we did not record antibiograms for the different species and have no data on rates of resistance, except for *S. aureus* and ESBL-producing Gram-negative bacteria. Infections caused by enterococci and anaerobes were more common in LowABR ICU patients which may explain the more frequent abdominal infections and use of metronidazole in these ICUs.

## Conclusions

Infections were more prevalent among patients admitted to ICUs in HighABR countries than in LowABR countries. Antibiotic therapy differed markedly, with broader spectrum antimicrobials being used more frequently in HighABR countries. ICU patients in HighABR countries were sicker and had longer ICU and hospital stays and higher crude ICU and hospital mortality rates, which could have a marked economical impact; however, after multivariate adjustment there were no differences in ICU or hospital mortality rates between the two groups of patients.

## Appendix: List of participating centres for this EPIC II substudy by country, alphabetically

*Denmark:* Århus University Hospital (H Betsch); Næstved Hospital (B Fogh); Rigshospitalet (K Espersen); Sygehus Fyn (K Jacobsen); Vejle Sygehus (P Berezowicz); *Finland:* Helsinki University Central Hospital (V Harjola); *Greece:* Ahepa University Hospital (E Sofianos); Athens University Medical School (A Armaganidis); Evangelismos Hospital (C Routsi); G.Papanikolaou (M Bitzani); General Hospital of Rethymno (A Chalkiadaki); Henry Dunant Hospital (A Michalopoulos); Hippokrateion Hospital Thessaloniki (E Mouloudi); Kat General Hospital (E Ioannidou); Kat Hospital (P Myrianthefs); Kat Hospital, Athens (D Koulenti); Konstantopoulio General Hospital (I Karampela); Lamia General Hospital (G Kyriazopoulos); Red Cross Hospital of Athens (K Mandragos); Thriassio Hospital of Eleusis (P Clouva-molyvdas); University Hospital of Ioannina (A Moraiti); University Hospital of Alexandroupolis (I Pneumatikos); University Hospital of Rion, Patras (K Filos); University Hospital of Thessaly (Larissa) (E Zakynthinos); University of Athens, Medical Shcool (A Kotanidou); Xanthi General Hospital (A Vakalos); *Israel:* Hadassah Medical Center (C Sprung); Haemek Medical Center (A Lev); Kaplan Medical Center (E Kishinevsky); Rabin Medical Center (J Cohen); Soroka Medical Center (S Sofer) *Italy:* A.O Niguarda (S Vesconi); A.O. Ospedale Di Circolo Di Busto Arsizio (S Greco); A.O. Treviglio-Caravaggio (M Borelli); Anestesia E Rianimazione 2 Prof.De Gaudio (P Cecilia); Arnas Ospedale Civico (M Sapuppo); ASL 10 (A Lazzero); ASL 10 Florence Hospital San Giovanni Di Dio (V Mangani); Azienda Ospedaliera Desenzano (N Petrucci); Azienda Ospedaliera Di Melegnano (M Minerva); Azienda Ospedaliera G. Rummo (E De blasio); Azienda Ospedaliera Polo Universitario San Paolo (S Marzorati); Azienda Ospedaliera Santa Maria Alle Scotte (R Rosi); Azienda Ospedaliera Universitaria P.Giaccone Policlinico (A Giarratano); Azienda Ospedaliera-Universitaria Udine (O Margarit); Azienda Ospedaliero -Universitaria (A Guberti); Azienda Ospedaliero-Universitaria S.M.Misericordia (S Scolz); Clinica San Gaudenzio (E Stelian); Fondazione IRCCS Policlinico San Matteo (V Emmi); Fondazione Ospedale Maggiore Policlinico, Mangiagalli Regina Elena (M Caspani); Fondazione Poliambulanza (A Rosano); H San Gerardo (C Abbruzzese); Hospital Panico Tricase (S Colonna); Humanitas Gavazzeni (R Ceriani); II Faculty of Medicne I University of Rome- Osp. S.Andrea (R De Blasi); S. Salvatore Hospital (L Panella); IRCCS Casa Sollievo Della Sofferenza (F Borrelli); Istituto Nazionale Tumori Regina Elena (P Lorella); KH Brixen (H Ruatti); Osepdali Riuniti Di Ancona (C Munch); Ospedale "Ca Foncello" - Treviso (Italia) (C Sorbara); Ospedale "Santa Croce" - ASL 8 (G Fiore); Ospedale Bufalini-Cesena (A Chieregato); Ospedale Di Circolo E Fondazione Macchi (V Conti); Ospedale Di Massa (A Guadagnucci); Azienda USL Piacenza (M Pizzamiglio); Ospedale Ferrarotto (M Locicero); Ospedale Maggiore Ausl Bologna (I Marri); Ospedale Maggiore Policlinico Milano (A Sicignano); Ospedale Maggiore Policlinico, Mangiagalli E Regina Elena, IRCCS Milano (V Conte); Ospedale Mugello Azienda Sanitaria Firenze (R Oggioni); Ospedale Niguarda Ca Granda, Milano (A De Gasperi); IRCCS Centro di Riferimento Oncologico della Basilicata (P De Negri); Ospedale Provinciale Pistoia (G Santagostino); Ospedale S. Gerardo (R Fumagalli); Ospedale San Raffaele (G Marino); Ospedele Vittorio Emanuele (G Castiglione); P.O. San Severo Asl Fg (D Sforza); S. Camillo Hospitql (N Giuseppe); San Martino Hospital (M Bassetti); Seconda Università Degli Studi Di Napoli (F Ferraro); Sesto San Giovanni Hospital (S Clementi); Teaching Hospital Careggi (D Alessandro); Terapia Intensiva - Aso S. Giovanni Battista Di Torino - Ospedale Molinette (P Cotogni, MV Ranieri); Università Cattolica (M Antonelli); Universita' Cattolica Del S. Cuore (L Martinelli); University-Hospital Careggi, Florence, (L Gianesello); University Hospital Policlinico Di Catania (A Gullo); University of Rome "La Sapienza" (A Morelli); UTI Trapianti (G Biancofiore); University of Udine (G Della Rocca) *Malta:* St Luke’s Hospital (M Borg); *Netherlands:* Academic Medical Center (A De Pont); Amphia Hospital (P Rosseel); Antoni Van Leeuwenhoek Ziekenhuis (J Ten Cate); Beatrix Zienhuis Rivas Zorggroep (G Van Berkel); Canisius Wilhelmina Ziekenhuis (S Corsten); Erasmus Mc University Medical Center (J Bakker); Hagaziekenhuis (J Vogelaar); Hofpoort Hospital Woerden (H Blom); Isala Clinics (H Kieft); Medical Center Leeuwarden (M Kuiper); Medisch Spectrum Twente (A Gille); Radboud University Nijmegen Medical Centre (P Pickkers); Rode Kruis Ziekenhuis (J Vet); Slingeland Ziekenhuis (J Ammann); Spaarneziekenhuis (S Den Boer); St. Antonius Ziekenhuis (R Wesselink); St.Elisabeth Hospital (B Speelberg); Twenteborg Hospital Almelo (C Pham); University Medical Center, Groningen (M Rodgers); University Hospital Maastricht (D Bergmans); Vu University Medical Center (J Groeneveld); *Norway:* Aker University Hospital (R Loevstad); St Olavs University Hospital (P Klepstad); Sykehuset Asker Og Bærum Hf (P Erno); Sykehuset I Vestfold Hf, Toensberg (A Junker); *Portugal:* Centro Hospitalar Alto Ave (A Bártolo); Centro Hospitalar Cova Da Beira (M Castelo-Branco Sousa); Centro Hospitalar Trás os Montes e Alto Douro (F Esteves); CHLO-Hospital S Francisco Xavier (A Martins); H S João (T Oliveira); Hospital CUF Infante Santo (P Ponce); Hospital Curry Cabral (L Mourão); Hospital da Luz (C Febra); Hospital de Egas Moniz (E Carmo); Hospital de S. José (V Lopes); Hospital de São Francisco Xavier (P Póvoa); Hospital de São José (A Rezende); Hospital Divino Espirito Santo (H Costa); Hospital do Litoral Alentejano (P Moreira); Hospital Dr. José Maria Grande, Portalegre (F Pádua); Hospital Fernando Fonseca (A Leite); Hospital Garcia de Orta (E Almeida); Hospital Geral de Santo António (M Alves); Hospital de Pulido Valente (A Sousa, L Telo); Hospital de S. João (C Dias, J Paiva); Hospital de São Bernardo (R Ribeiro); Hospital de São Sebastião, EPE (P Amaro); Hospital Geral de Sto Antánio (A Carneiro); Hospital de St. António dos Capuchos (R Moreno, Ricardo Matos, Susana Afonso); Instituto Português de Oncologia de Lisboa (M Bouw); Hospital de St Maria (C França); *Spain:* Althaia (O Rubio); Bellvitge University Hospital (R Mañez); Centro Medico Delfos (M Burgueño Campiñez); Clinica Moncloa (M Alvarez); Clinica Rotger (R Jorda); Clinica Santa Elena (E Naveira-Abeigón); Clinica Universitaria de Navarra (P Monedero); Complejo Hospitalario de Pontevedra (E Alemparte-Pardavila); Fundacion Hospital Alcorcon (S Garcia del Valle); Fundacion Jimenez Diaz (C Perez Calvo); H Vall Hebron (M Palomar); H.U. Virgen de Las Nieves- H. Traumatología Y Rehabilitación (F Guerrero); Hospital "Virgen de La Concha" - Zamora (A Caballero Zirena); Hospital Arnau de Vilanova (M Arribas); Hospital Can Misses (E Bustamante Munguira); Hospital Casa de Salud (J Ruiz); Hospital Central de Asturias (L Iglesias); Hospital Clinic (E Zavala); Hospital Clinic de Barcelona (M Valencia); Hospital Clinico San Carlos (A Blesa Malpica); Hospital Clinico San Carlos (F Martinez-Sagasti, M Nieto); Hospital Clinico Universitario (G Aguilar); Hospital Clinico Universitario de Santiago (F Martinon-Torres); Hospital Comarcal Vinaros (C Lorente); Hospital de Navarra (J Insausti); Hospital de Antequera (R Vegas Pinto); Hospital de Basurto (I Santos); Hospital de Fuenlabrada (A Escriba); Hospital de Galdakao (P Olaechea); Hospital de La Merced (E Muñoz); Hospital de Manacor (E Antón Caraballo); Hospital de Mostoles (P Galdos-Anuncibay); Hospital de Sagunto (V Lopez Camps); Hospital de Tortosa Verge de La Cinta (F Esteban-Reboll); Hospital del Sas de Jerez (A Estella); Hospital Donostia (L Bocero); Hospital Dr Peset (A Ibañez); Hospital G. Yagüe (L Pueyo); Hospital General (L María Jesús); Hospital General de Asturias (L Iglesias); Hospital General de Ciudad Real (J Silva); Hospital General de Granollers (P Garro); Hospital General de La Palma (L Ramos-gómez); Hospital General de L'Hospitalet (A Rovira); Hospital General de Vic (M Martin Delgado); Hospital General Salud "Obispo Polanco" (J Monton Dito); Hospital General Universitario de Albacete (F Garcia); Hospital General Universitario de Alicante (J Navarro); Hospital General Universitario de Elche (J Latour-Perez); Hospital General Universitario de Guadalajara (A Albaya); Hospital General Universitario Gregorio Marañon (A Bustinza); Hospital Gran Canaria "Dr Negrín" (J Sole-violán); Hospital Marques de Valdecilla (P Ugarte Peña); Hospital Maz (I Yuste); Hospital Parque San Antonio (J De Rojas Román); Hospital Sabadell (J Vallés); Hospital Sant Joan de Déu (E Esteban); Hospital Sant Pau (E Quintana Tort-Martorell); Hospital Santa María del Rosell (M Moreno); Hospital Santa Maria Madre-Complejo Hospitalario de Ourense (V López Ciudad); Hospital Santiago Apostol (A Manzano Ramirez); Hospital Sevilla-Aljarafe (J Sánchez-Olmedo); Hospital Son Llàtzer (M Borges); Hospital Terrassa (J Amador Amerigo); Hospital Torrecardenas (F Guerrero Gomez); Hospital Universitaio 12 de Octubre (J Montejo González); Hospital Universitari de Girona Doctor Josep Trueta (J Sirvent); Hospital Universitari Germans Trias I Pujol (E Mesalles Sanjuan); Hospital Universitario Arnau de Vilanova (F Barcenilla-Gaite); Hospital Universitario de Canarias (N Serrano); Hospital Universitario de Getafe (E Cerdá); Hospital Universitario de Valme (A Lesmes Serrano); Hospital Universitario Doce de Octubre (C Garcia-Fuentes); Hospital Universitario Infanta Crsitina (J Macias Pingarrón); Hospital Universitario Nªsra de Candelaria (E Espinosa); Hospital Universitario Principe de Asturias (M Sanchez Garcia); Hospital Universitario Reina Sofía, Murcia (F Felices); Hospital Universitario Virgen de La Victoria (M de la Torre-Prados); Hospital Univesitario Puerto Real (H Maria Jesus); Hospital Valle del Nalon (V Luis); Hospital Virgen Arrixaca (R Jara); Hospital Xanit Internacional (M Briones Lopez); Hospital Xeral Cies (P Posada); Hu La Paz (B Galvan); Hu La Paz (F Mariscal); Joan Xxiii University Hospital (J Rello); Morales Meseguer (B Gil); Puerta del Mar Universitary Hospital (R Sierra); Rio Hortega University Hospital (J Rico-Feijoo); San Pedro de Alcantara Hospital (C Corcobado Márquez); Servicio Navarro de Salud.Hospital Virgen del Camino (J Izura); Uci H. U. Salamanca (J González); Universitary Hospital Dr. Peset (J Soto Ibáñez); *Sweden:* Anestesikliniken (P Petersen); Centralsjukhuset Karlstad (L Johansson); University Hospital, Linköping (H Blomqvist, B Peterzén, N Wyon); Göteborg (I Lindström); Kärnsjukhuset Skövde (A Paulsson); Karolinska University Hospital Huddinge (C Agvald-Ohman); Karolinska University Hospital, Solna (J Petersson); Lund University Hospital (H Friberg); Malmoe University Hospital (V Einar); Op/Iva Kliniken (F Hammarskjöld); Ostersund Hospital (M Schindele); Ostra Hospital, Göteborg (S Arvidsson); Sahlgrenska University Hospital (J Sellgren); Söder Hospital (Södersjukhuset) (J Hulting); Sodersjukhuset (J Häggqvist); Sollefteå Hospital (J Rudenstam); Sunderby Hospital (D Lind); The Queen Silvia Children’s Hospital (E Kokinsky); Thoracic Intensive Care, Karolinska Hospital (A Owall); Umeå University Hospital (S Jacobson); University Hospital (H Stiernstrom); University Hospital of Örebro (A Nydahl); *Turkey:* Acibadem Kadikoy Hospital (K Atalan); Acibadem Bakirkoy Hospital (C Ates); Acibadem Bursa Hospital (A Kahveci); Acibadem Kozyatagi Hospital (H Fistikci); Ankara Univercity (A Kaya); Ankara University Medical Faculty, Ibni Sina Hospital (E Ozgencil); Ataturk University Medical Faculty (M Kizilkaya); Dicle University Medical School (M Bosnak); Dokuz Eylul University (H Bodur); Dokuz Eylul University School of Medicine (M Akan); Erciyes University Medical Faculty (M Guven); Gazi University School of Medicine (M Turkoglu); Hacettepe University Hospital (A Topeli); Inonu University Medical Faculty (T Togal); Istanbul Faculty of Medicine (N Uzel); Istanbul Medical Faculty (I Akinci, N Cakar, S Tugrul); Istanbul University Cerrahpasa Medical School (O Demirkiran); Izmir Ataturk Training and Resarch Hospital (T Adanir); Memorial Hospital (K Dogruer); Okmeydani Teaching & Research Hospital (A Turkmen); Okmeydani Teaching & Research Hospital (H Guven); Ondokuz Mayis University, Medical Faculty (F Ulger); Selcuk University Meram Faculty of Medicine (S Kocak).

## Electronic supplementary material

Additional file 1: Sites of infection.(PDF 93 KB)

Additional file 2: Use of antimicrobials as prophylaxis in all patients.(PDF 100 KB)
