# Supplementary material for: Infections, antibiotic treatment and mortality in patients admitted to ICUs in countries considered to have high levels of antibiotic resistance compared to those with low levels
Source: BMC Infect Dis. 2014 Sep 22;14:513. doi: 10.1186/1471-2334-14-513 (PMC4181425; doi:10.1186/1471-2334-14-513)
Supplement: Supplementary file 1 — Additional file 1: Sites of infection.(PDF 93 KB) [file 12879_2014_3832_MOESM1_ESM.pdf]

Infections, antibiotic treatment and mortality in patients admitted to ICUs in countries considered to have high levels of antibiotic resistance compared to those with low levels

Håkan Hanberger[1], Massimo Antonelli[2], Martin Holmbom[1], Jeffrey Lipman[3], Peter Pickkers[4], Marc Leone[5], Jordi Rello[6], Yasser Sakr[7], Sten M Walther[8], Philippe Vanhems[9], Jean-Louis Vincent[10]
for the EPIC II Group of Investigators

Additional file 1: Sites of infection

Site/source of infection, n (%)	Patients from lowABR countries (n=255)	Patients from highABR countries (n=1187)	p-value
Respiratory	154 (60.4)	761 (64.1)	0.263
Abdominal	62 (24.3)	218 (18.4)	0.029
Blood	41 (16.1)	233 (19.6)	0.029
Renal / Urinary	22 (8.6)	148 (12.5)	0.190
Skin	15 (5.9)	67 (5.6)	0.084
Catheter	12 (4.7)	76 (6.4)	0.882
CNS	4 (1.6)	35 (2.9)	0.304
Other	32 (12.5)	65 (5.5)	<0.001

Patients could have more than one site of infection. CNS, central nervous system.
