# Supplementary material for: Infections, antibiotic treatment and mortality in patients admitted to ICUs in countries considered to have high levels of antibiotic resistance compared to those with low levels
Source: BMC Infect Dis. 2014 Sep 22;14:513. doi: 10.1186/1471-2334-14-513 (PMC4181425; doi:10.1186/1471-2334-14-513)
Supplement: Supplementary file 2 — Additional file 2: Use of antimicrobials as prophylaxis in all patients.(PDF 100 KB) [file 12879_2014_3832_MOESM2_ESM.pdf]

**Additional file 2:** Use of antimicrobials as prophylaxis in all patients

| | LowABR ICU patients (n=183) | HighABR ICU patients (n=489) | P value |
|---|---|---|---|
| **Antimicrobial, n (%)** | | | |
| Cephalosporins (all) | 127 (22.8) | 301 (13.7) | <0.001 |
| *Cefazolin* | 55 (9.9) | 158 (7.2) | 0.034 |
| *Cefuroxime* | 44 (7.9) | 37 (1.7) | <0.001 |
| *Ceftazidime* | 3 (0.5) | 20 (0.9) | 0.390 |
| *Cefepime/Cefpirome* | 0 (0) | 1 (0) | 0.615 |
| *Other cephalosporins* | 26 (4.7) | 85 (3.9) | 0.388 |
| **Penicillins (all)** | 41 (7.3) | 205 (9.3) | 0.148 |
| *Benzylpenicillin* | 2 (0.4) | 1 (0) | 0.045 |
| *Ampicillin* | 1 (0.2) | 39 (1.8) | 0.005 |
| *Amoxycillin+Clavulanate* | 4 (0.7) | 86 (3.9) | <0.001 |
| *Piperacillin+Tazobactam* | 5 (0.9) | 72 (3.3) | 0.002 |
| *Oxa-/Cloca-/Flucloxacillin* | 27 (4.8) | 1 (0) | <0.001 |
| *Unspecified penicillins* | 2 (0.4) | 8 (0.4) | 0.987 |
| *Temocillin* | 0 (0) | 0 (0) | 0.000 |
| **Other β-lactams (all)** | 4 (0.7) | 53 (2.4) | 0.012 |
| *Imipenem/Meropenem* | 4 (0.7) | 50 (2.3) | 0.018 |
| *Aztreonam* | 0 (0) | 1 (0) | 0.615 |
| *Unspecified β-lactams* | 0(0) | 2 (0.1) | 0.477 |
| **Aminoglycosides (all)** | 76 (13.6) | 43 (2) | <0.001 |
| *Amikacin* | 1 (0.2) | 9 (0.4) | 0.421 |
| *Tobramycin* | 64 (11.5) | 17 (0.8) | <0.001 |
| *Other aminoglycosides* | 25 (4.5) | 17 (0.8) | <0.001 |
| **Quinolones (all)** | 3 (0.5) | 53 (2.4) | 0.005 |
| *Ciprofloxacin* | 2 (0.4) | 22 (1) | 0.146 |
| *Other quinolones* | 1 (0.2) | 31 (1.4) | 0.016 |
| **Glycopeptides (all)** | 2 (0.4) | 65 (2.9) | <0.001 |
| *Vancomycin* | 2 (0.4) | 38 (1.7) | 0.016 |
| *Other glycopeptides* | 0 (0) | 27 (1.2) | 0.009 |
| **Macrolides (all)** | 4 (0.7) | 6 (0.3) | 0.118 |
| *Erythromycin* | 3 (0.5) | 1 (0) | 0.006 |
| *Other macrolides* | 1 (0.2) | 5 (0.2) | 0.829 |
| **Other antibiotics (all)** | 92 (16.5) | 92 (4.2) | <0.001 |
| *Metronidazole* | 23 (4.1) | 51 (2.3) | 0.018 |
| *Cotrimoxazole* | 4 (0.7) | 13 (0.6) | 0.732 |
| *Oxazolidinone* | 0 (0) | 7 (0.3) | 0.183 |
| *Unspecified antibiotics* | 66 (11.8) | 24 (1.1) | <0.001 |
| **Antifungals (all)** | 74 (13.3) | 57 (2.6) | <0.001 |
| *Fluconazole* | 5 (0.9) | 33 (1.5) | 0.276 |
| *Amphotericin B* | 63 (11.3) | 13 (0.6) | <0.001 |
| *Amphotericin Lipid Complez* | 2 (0.4) | 2 (0.1) | 0.137 |
| *Caspofungin* | 2 (0.4) | 7 (0.3) | 0.880 |
| *voriconazole* | 0 (0) | 1 (0) | 0.615 |

| | | | |
|---|---|---|---|
| *Other antifungals* | 2 (0.4) | 2 (0.1) | 0.137 |
| **Antiviral (all)** | 3 (0.5) | 7 (0.3) | 0.440 |
